# The *Epichloë festucae* Antifungal Protein *Efe*-AfpA Has Activity against Numerous Plant Pathogens

**DOI:** 10.3390/microorganisms11040828

**Published:** 2023-03-24

**Authors:** Patrick A. Fardella, Bruce B. Clarke, Faith C. Belanger

**Affiliations:** Department of Plant Biology, Rutgers University, New Brunswick, NJ 08901, USA

**Keywords:** antifungal protein, fungal endophyte, fungal plant pathogens

## Abstract

Fungal plant pathogens can present major problems for most crop species. Currently, control of fungal diseases relies heavily on the use of fungicides. However, there are problems associated with fungicide use, including potential toxicity to non-target organisms and the development of resistance in the target fungus. New strategies are being sought to reduce fungicide use. One area of active research is the potential use of antifungal proteins from various fungal species as alternatives or complements to traditional fungicides. An antifungal protein, *Efe*-AfpA, from the fungal endophyte *Epichloë festucae* was previously found to protect plants from the pathogen *Clarireedia jacksonii*, the causal agent of dollar spot disease. Here we report that *Efe*-AfpA also has inhibitory activity against other important plant pathogens. These results suggest that it may be possible to develop *Efe*-AfpA as a biofungicide to target a broad range of destructive plant pathogens.

## 1. Introduction

Management of fungal plant pathogens is challenging for many crops. Current management strategies rely heavily on the use of fungicides, which are considered critical for global food security [1,2]. Fungicides are also used heavily on amenity crops such as turfgrasses [3]. Although deemed critical for disease management, there are several problems associated with fungicide use, including potential toxicity to non-target organisms and the development of resistance in the target fungus [1]. Strategies to reduce fungicide usage include the breeding of more disease-tolerant cultivars [4,5] as well as genetic modification of plants to enhance disease resistance [6]. Improved cultural management strategies can also help to improve disease control and reduce fungicide usage [7].

Another strategy is the development of alternatives or complements to synthetic fungicides that have reduced potential for causing toxicities to the environment or non-target organisms. This approach can involve the application of biological control organisms or products derived from living organisms to plants to reduce disease severity [8]. One area of active research is the potential use of antifungal proteins from various fungal species as biofungicides. The most extensively researched antifungal proteins are PAF and PAFB from *Penicillium chrysogenum*, and AFP and NFAP from *Aspergillus* spp. [9,10,11,12]. These proteins have been tested against a variety of fungi, yeasts, and bacteria to determine their efficacy in inhibiting growth, showing both stark differences and striking similarities in their effectiveness [10,13,14,15]. This underscores the importance of continuing to test these and additional antifungal proteins to determine their repertoire of sensitive microorganisms. This information would allow for the selection of appropriate antifungal proteins to help control specific plant pathogens [16].

We are interested in the potential development of an antifungal protein from the endophytic fungus *Epichloë festucae*, which infects the grass strong creeping red fescue (*Festuca rubra* subsp. *rubra*), as a possible alternative or complement to fungicides for the control of important plant pathogens. Strong creeping red fescue is a commercially important turfgrass species [17]. *Epichloë* spp. are common endophytic symbionts of many cool-season grass species, often conferring insect resistance to the hosts due to the production of toxic alkaloids [18]. In addition to insect resistance, *E. festucae* infection is known to confer field-level disease resistance in strong creeping red fescue against both dollar spot disease (*Clarireedia jacksonii*) and red thread disease (*Laetisaria fuciformis*) [19,20]. This endophyte-mediated disease resistance is unique to the *E. festucae*/fine fescue symbiosis and has not been seen in other *Epichloë* spp./commercial grass species interactions [20,21,22]. The genome of *E. festucae* isolates that infect strong creeping red fescue contains a gene encoding an antifungal protein [23]. The *E. festucae* antifungal protein gene is designated *Efe-afpA* and the protein is designated *Efe*-AfpA [24]. Such a gene is not present in the genomes of most *Epichloë* spp. for which whole genome sequencing is available, including other strains of *E. festucae* [21,23] This suggested that the *E. festucae* antifungal protein is a likely factor in the endophyte-mediated disease resistance observed in strong creeping red fescue. To validate the presumption that *Efe*-AfpA was a major component of this endophyte-mediated disease resistance, *Efe*-AfpA was produced in a *Pe. chrysogenum* expression system and tested against *Cl. jacksonii*. Purified *Efe*-AfpA inhibited the growth of *Cl. jacksonii* in culture and direct application to *Cl. jacksonii*-inoculated plants reduced the severity of disease symptoms on endophyte-free strong creeping red fescue and creeping bentgrass (*Agrostis stolonifera*) [25].

These results support the original hypothesis that *Efe*-AfpA is a major contributor to the dollar spot resistance seen in endophyte-infected strong creeping red fescue plants and suggested that *Efe*-AfpA may have potential as a biofungicide. It was therefore of interest to determine if *Efe*-AfpA also had activity against other plant pathogens. The objective of this work was to assay *Efe*-AfpA activity on important plant fungal pathogens in culture. We also assayed the *Pe. chrysogenum* antifungal protein PAF since we previously found differences between the activities of *Efe*-AfpA and PAF against *Cl. jacksonii* [25]. In particular, we focused on historically and economically important plant pathogens and major turfgrass pathogens, as *Efe*-AfpA is from a turfgrass endophyte. *Efe*-AfpA did have inhibitory activity against several other plant pathogens, expanding its potential usefulness beyond *Cl. jacksonii*.

## 2. Materials and Methods

### 2.1. Fungi and Culture Conditions

*Pe. chrysogenum paf*, an isolate over-expressing *paf*, and *Pe. chrysogenum^Efe^*^-AfpA^, an isolate expressing *Efe-afpA*, were maintained on PcMM (*Penicillium chrysogenum* Minimal Media) [25,26] supplemented with 200 μg mL^−1^ nourseothricin and 0.6 μg mL^−1^ pyrithiamine. All other fungi were maintained on potato dextrose agar (PDA) (HiMedia Laboratories, Mumbai, India). To generate conidia for *Pyricularia oryze*, a fungal plug grown on PDA was subcultured onto ryegrass-amended plates and grown for at least 2 weeks. Ryegrass-amended plates were made by autoclaving ryegrass clippings, approximately 20 g in 500 mL of distilled water twice. The clippings were removed by filtering through cheesecloth. The volume of the filtered solution was brought back up to 500 mL with distilled water, agar was added to a final concentration of 1.5%, and the solution was autoclaved.

### 2.2. Purification of Efe-AfpA from Pe. chrysogenum^Efe-AfpA^ and PAF from Pe. chrysogenum paf

The protein purification method was previously described in detail [25]. Briefly, for purification of *Efe*-AfpA 2 × 10^8^ conidia of *Pe. chrysogenum^Efe-AfpA^* were added to 200 mL *Aspergillus nidulans* Complete Media and shaken at 200 rpm for 48 h. The mycelium was then harvested by filtering the culture through cheesecloth, which was then resuspended in 200 mL PcMM and incubated for 72 h while shaking at 200 rpm. The culture supernatant was collected by filtering through cheesecloth to remove mycelia, and any excess debris was pelleted by centrifugation at 10,000 rpm for 10 min. The cleared culture supernatant was applied to a carboxymethyl cellulose (CMC52) (Biophoretics, Sparks, NV, USA) column pre-equilibrated with 10 mM NaPO_4_, 25 mM NaCl, 0.15 mM EDTA, pH 6.6. The column was then washed with excess buffer and *Efe*-AfpA was eluted with increasing salt concentrations from 0.1 to 0.5 M NaCl. Similarly, for purification of PAF, 2 × 10^8^ conidia of *Pe. chrysogenum paf* was added to 200 mL PcMM and shaken for 72 h at 200 rpm. The culture supernatant was then processed as described above for *Efe*-AfpA.

Eluted fractions from both purifications were evaluated for the presence of the protein utilizing their respective molecular weights and extinction coefficients at A_280_ measured by using a Nanodrop ND-1000 spectrophotometer (Thermo Fisher Scientific, Waltham, MA, USA). *Efe*-AfpA molecular weight: 6.278 kDa, extinction coefficient: 5220 M^−1^ cm^−1^. PAF molecular weight: 6.25 kDa, extinction coefficient: 4845 M^−1^ cm^−1^. Protein-containing fractions were filtered through a 30 kDa Amicon^®^ Ultra-15 Centrifugal Filter (MilliporeSigma, Burlington, MA, USA) to remove high molecular weight proteins. The proteins were then concentrated and desalted on a 3 kDa Amicon^®^ Ultra-15 Centrifugal Filter with sterile distilled water. The protein samples were finally sterilized by filtering through a 0.2 μm polyethersulfone syringe filter (Corning Inc., Corning, NY, USA).

### 2.3. Antifungal Activity Assays

For fungi that easily produced spores (*B. cinerea*, *Co. graminicola*, *F. graminearum* PH1, *Py. oyzae*), antifungal activities of *Efe*-AfpA and PAF were assayed in 24 and 96-well plates. For the 24-well plate activity assays, spores were harvested in Spore Buffer (0.9% NaCl, 0.01% Tween 20), washed twice in Spore Buffer, washed once in sterile distilled water, and resuspended in sterile distilled water. Spores were counted using a hemocytometer and diluted to 2 × 10^5^ conidia mL^−1^. Five μL of spores was plated onto 500 μL of PDA amended with increasing concentrations of *Efe*-AfpA or PAF (0, 0.75, 1.5, 3, 6, 12, 25, 500, 100 μg mL^−1^) in a 24-well plate. Plates were incubated for 72 to 96 h, depending on the growth rate of the fungus. Assays were performed in duplicate and the experiment was completed twice.

For 96-well activity assays, spores were harvested in Spore Buffer, washed twice in Spore Buffer, washed once in 2 × low cation media (LCM) (1 × LCM is 2 g L^−1^ glucose, 0.1 g L^−1^ yeast extract, 0.05 g L^−1^ peptone), and resuspended in fresh 2 × LCM. Spores were counted on a hemocytometer and diluted to 2 × 10^4^ conidia mL^−1^ with 2 × LCM. One hundred μL of spores and 100 μL of water containing increasing concentrations of *Efe*-AfpA or PAF were incubated in each well to final concentrations of 0, 0.3, 0.6, 1.2, 5, 10, 20, 30, 40, 50, and 100 μg mL^−1^ antifungal protein. Plates were incubated at room temperature for 24 to 48 h, depending on how fast the fungi grew. Growth was monitored at A_620_ using a microtiter plate reader (Absorbance 96, Byonoy GmbH, Hamburg, Germany). Optical density at A_620_ was measured at 0 h and subtracted from subsequent readings to correct for background absorbance. The corrected absorbance of untreated control conidia was considered 100% growth. The percent inhibition by each protein was calculated by comparing the conidia growth of the treated samples to the untreated samples. Wells were visualized microscopically (EVOS M5000, Invitrogen, Waltham, MA, USA). Each treatment had 3 replicates and the experiment was completed twice.

Fungi that did not produce spores easily (*Cr. parasitica* Ep155, *L. fuciformis*, *R. solani*) had their sensitivity to *Efe*-AfpA and PAF determined using fungal plugs. These fungi were grown from 4 to 10 days on PDA, depending on the growth rate of the fungus, prior to being subcultured. A 5 mm plug of each fungus was placed in the center of 8 mL PDA plates amended with increasing concentrations of *Efe*-AfpA or PAF (0, 0.5, 1, 10, 20, 30, 40, 50, 100 μg mL^−1^). Cross-sectional fungal diameter was measured, and plates were photographed daily. Each treatment had three replicates and the experiment was completed twice.

## 3. Results

### 3.1. Botrytis cinerea

**Figure 1:**
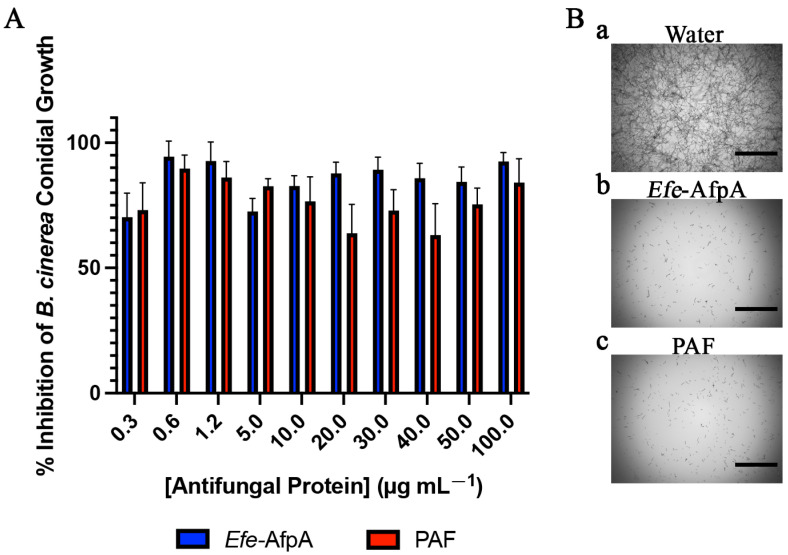
Activity of *Efe*-AfpA and PAF against *B. cinerea* conidial growth. (**A**). Growth inhibition of *B. cinerea* conidia treated with increasing concentrations of either *Efe*-AfpA or PAF incubated at room temperature for 30 h. The data presented are the means and standard deviations of three replicates. (**B**) Microscopy of *B. cinerea* conidia treated with water or 0.6 μg mL^−1^ of either *Efe*-AfpA or PAF. Bars are 750 μm.

*Botrytis cinerea* is a necrotrophic ascomycete responsible for gray mold on over 200 crop species worldwide. Although it causes disease issues in the field and in greenhouses, it is also a considerable post-harvest problem because the fungus can remain quiescent for extended periods of time before becoming active [27]. *B. cinerea* was listed as the second most important fungal plant pathogen and causes between $10 to $100 billion in losses annually worldwide [28,29]. Previous work reported variable activity of PAF against *B. cinerea*, which could be due to differences in experimental assays used [30,31,32]. In the current study, *Efe*-AfpA had a minimal inhibitory concentration (MIC) against *B. cinerea* of 0.6 μg mL^−1^ (Figure 1 and Figure S1, Table 1). MIC is defined as the minimal concentration resulting in at least 90% inhibition of the growth of the target organism. PAF did have activity against *B. cinerea* but its activity did not result in 90% inhibition at any of the concentrations tested (Figure 1, Table 1).

### 3.2. Colletotrichum cereale

**Figure 2:**
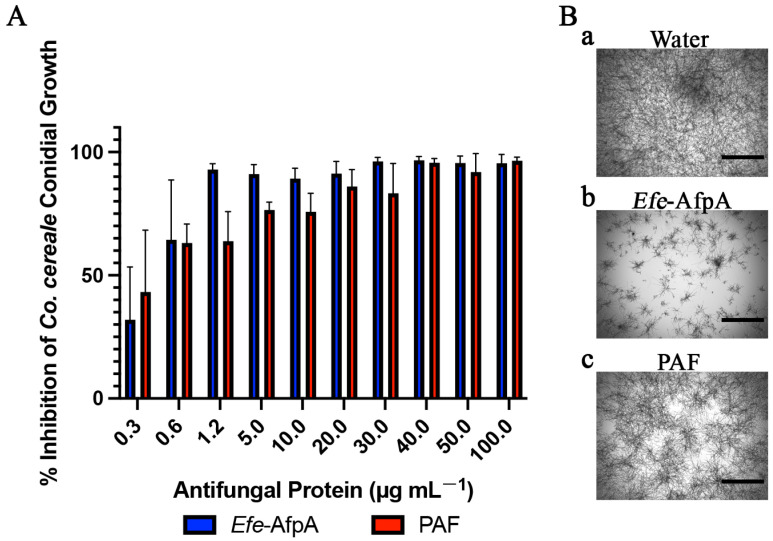
Activity of *Efe*-AfpA and PAF against *Co. cereale* conidial growth. (**A**) Growth inhibition of *Co. cereale* conidia treated with increasing concentrations of either *Efe*-AfpA or PAF incubated at room temperature for 48 h. The data presented are the means and standard deviations of three replicates. (**B**) Microscopy of *Co. cereale* conidia treated with water or 1.2 μg mL^−1^ of either *Efe*-AfpA or PAF. Bars are 750 μm.

*Colletotrichum* spp. are common and destructive pathogens on many plant species and are particularly destructive on most agricultural crops. They are ranked the eighth most important fungal pathogens of plants [28]. *Co. cereale* is responsible for anthracnose on turfgrasses such as *Poa annua* and *Agrostis* species, where the disease occurs as either a foliar blight or basal rot [33]. Both *Efe*-AfpA and PAF had activity against *Co. cereale* that resulted in greater than 90% inhibition, with MIC concentrations of 1.2 and 40 μg mL^−1^, respectively (Figure 2 and Figure S2, Table 1).

### 3.3. Cryphonectria parasitica 

**Figure 3:**
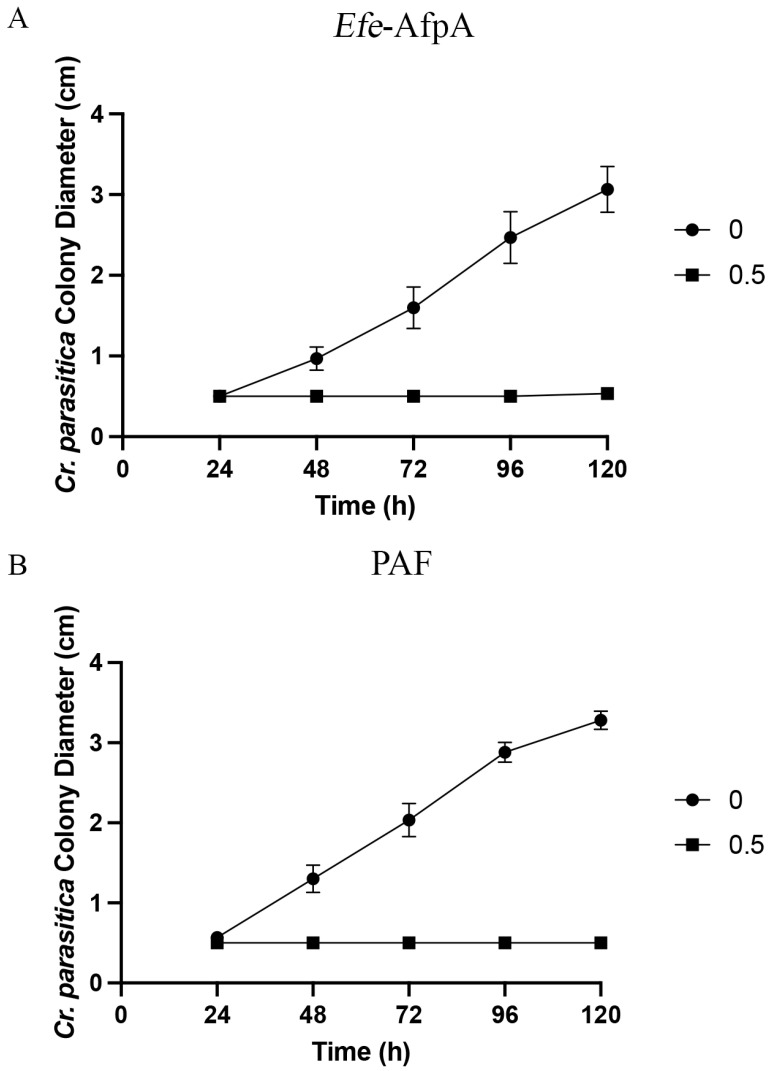
Activity of (**A**) *Efe*-AfpA and (**B**) PAF against *Cr. parasitica* mycelial growth. *Cr. parasitica* mycelial plugs were subcultured onto PDA plates amended with 0.5 μg mL^−1^ of *Efe*-AfpA or PAF. The colony diameters were measured daily. The data presented are the means and standard deviations of three replicates.

*Cryphonectria parasitica* EP155 is the causal agent of chestnut blight, which nearly eliminated the American chestnut tree (*Castanea dentata*) worldwide [34]. Current methods of alleviating the disease and restoring the American chestnut to its native range include the development of interspecific hybrids, backcross breeding, and genetic engineering [35]. Since *Cr. parasitica* produces spores slowly, taking 3 to 4 weeks [36], the inhibitory activity of the antifungal proteins was determined in 8 mL agar plate assays using fungal mycelium plugs. Both *Efe*-AfpA and PAF were active against *Cr. parasitica*, with the same MIC value of 0.5 μg mL^−1^ (Figure 3, Figures S3 and S4, Table 1). Since there was nearly complete inhibition at the lowest concentration tested, 0.5 μg mL^−1^, higher concentrations are not shown in the graphs in Figure 3.

### 3.4. Fusarium graminearum 

**Figure 4:**
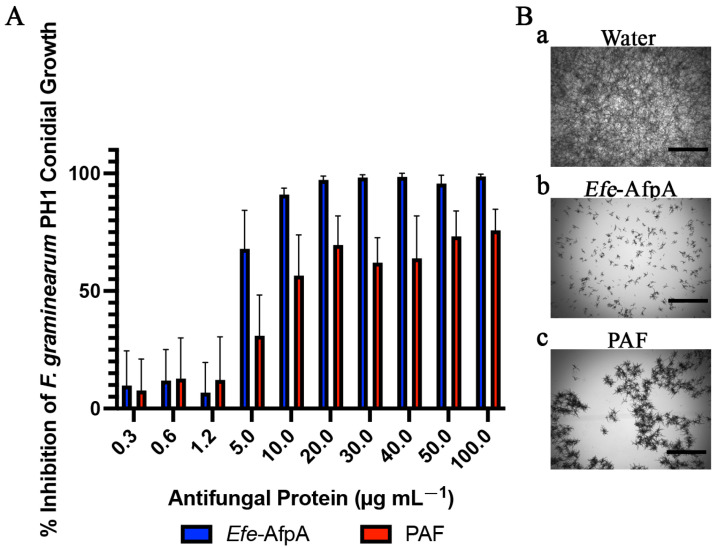
Activity of *Efe*-AfpA and PAF against *F. graminearum* PH1 conidial growth. (**A**) Growth inhibition of *F. graminearum* conidia treated with increasing concentrations of either *Efe*-AfpA or PAF incubated at room temperature for 30 h. The data presented are the means and standard deviations of three replicates. (**B**) Microscopy of *F. graminearum* conidia treated with water or 10 μg mL^−1^ of either *Efe*-AfpA or PAF. Bars are 750 μm.

*Fusarium graminearum* PH1 causes both Fusarium head blight on wheat as well as barley and Fusarium stalk and ear rot on maize and was considered the fourth most important fungal plant pathogen [28,37]. In addition to its destructive impact on wheat and barley, *F. graminearum* produces mycotoxins detrimental to human and animal health, such as deoxynivalenol (DON) [38]. Both *Efe*-AfpA and PAF had activity against *F. graminearum*, but only *Efe*-AfpA had an activity that resulted in greater than 90% inhibition, with a MIC of 10 μg mL^−1^ (Figure 4 and Figure S5, Table 1).

### 3.5. Pyricularia oryzae 

**Figure 5:**
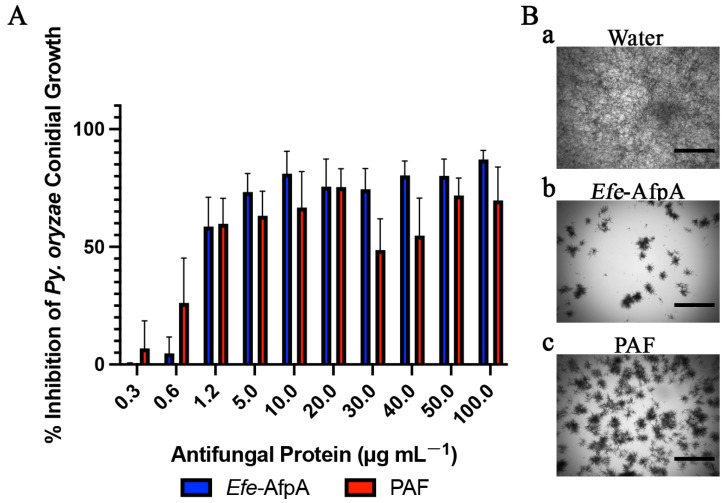
Activity of *Efe*-AfpA and PAF against *Py. oryzae* conidial growth. (**A**) Growth inhibition of *Py. oryzae* conidia treated with increasing concentrations of either *Efe*-AfpA or PAF incubated at room temperature for 48 h. The data presented are the means and standard deviations of three replicates. (**B**) Microscopy of *Py. oryzae* conidia treated with water or 100 μg mL^−1^ of either *Efe*-AfpA or PAF. Bars are 750 μm.

*Pyricularia oryzae* (previously *Magnaporthe oryzae*) is the causal agent of rice blast and was voted the number one fungal plant pathogen from a scientific and economic perspective [28,39]. Rice (*Oryza sativa*) is an economically and agriculturally important crop that feeds about half the world’s population, and from 10 to 30% of yield can be lost annually due to rice blast disease [40]. *Py. oryzae* also causes gray leaf spot of many genera of turfgrass including *Cynodon, Eremochloa*, *Festuca*, *Lolium*, *Paspalum*, *Pennisetum*, and *Stenotaphrum* [33]. Gray leaf spot was also recently identified on the turfgrass *Festuca brevipila* (hard fescue), which had not previously been reported to be sensitive to *Py. oryzae* [41]. As the pathogen presents problems on both rice and turf, new mechanisms of plant protection are needed. The well-studied antifungal proteins AFP from *A. niger* and PAF from *Pe. chrysogenum* have been tested against *Py. oryzae.* AFP was highly effective at completely inhibiting growth at the low concentration of 4 μM but PAF was less effective, requiring a concentration greater than 200 μg mL^−1^ (approximately 32 μM) to completely inhibit growth [32,42]. Here, both *Efe*-AfpA and PAF did have activity against *Py. oryzae*, but their activities did not result in 90% inhibition at any of the concentrations tested (Figure 5 and Figure S6, Table 1).

### 3.6. Laetisaria fuciformis and Rhizoctonia solani

**Figure 6:**
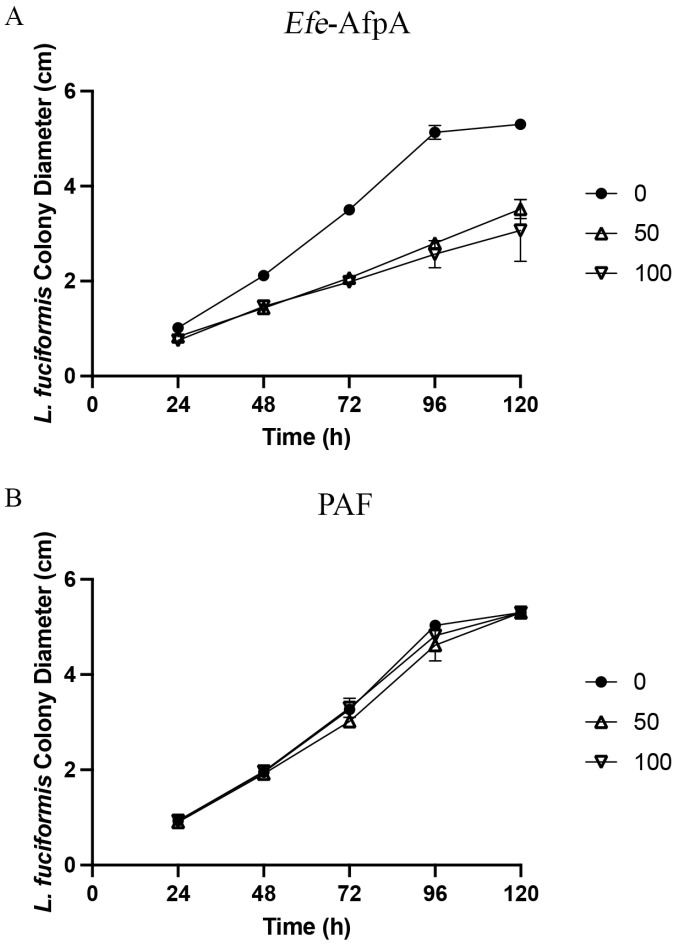
Activity of (**A**) *Efe*-AfpA and (**B**) PAF against *L. fuciformis* mycelial growth. *L. fuciformis* mycelial plugs were subcultured onto PDA plates amended with increasing concentrations of *Efe*-AfpA or PAF. The colony diameters were measured daily. The data presented are the means and standard deviations of three replicates.

**Figure 7:**
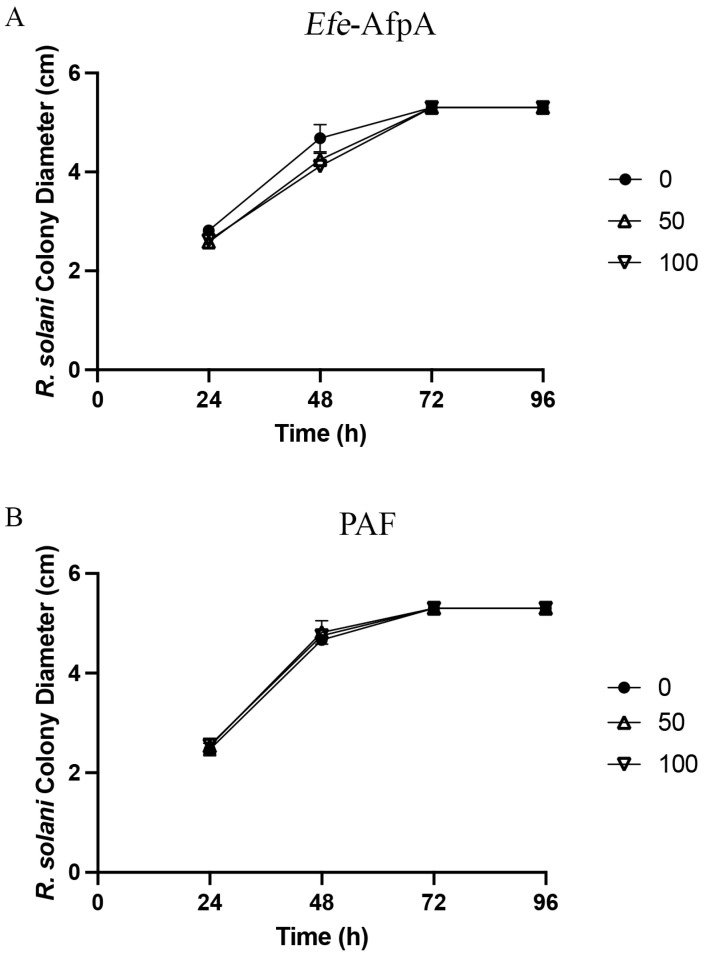
Activity of (**A**) *Efe*-AfpA and (**B**) PAF against *R. solani* mycelial growth. *R. solani* mycelial plugs were subcultured onto PDA plates amended with increasing concentrations of *Efe*-AfpA or PAF. The colony diameters were measured daily. The data presented are the means and standard deviations of three replicates.

*Laetisaria fuciformis* causes red thread of turfgrasses, and is particularly damaging to *Lolium perenne* (perennial ryegrass) and *Festuca rubra* [33]. *F. rubra* has been shown to have enhanced tolerance to red thread when infected by the fungal endophyte *E. festucae* [19]. *Rhizoctonia* spp. cause several diseases in different types of turfgrass species. *R. solani* causes brown patch in cool-season turf and large patch in warm-season turf [33]. *Efe*-AfpA had some activity against *L. fuciformis* but none against *R. solani* (Figure 6 and Figure 7, Table 1, Figures S7–S10). PAF was ineffective against both *L. fuciformis* and *R. solani.* Only the two highest concentrations tested are shown in Figure 6 and Figure 7 since there was negligible inhibitory activity at the lower concentrations.

## 4. Discussion

Here, we evaluated the antifungal activity of *Efe*-AfpA and PAF against some important fungal plant pathogens in culture. Both proteins were effective at inhibiting the growth of some of the pathogens, but there were some clear differences in their activity levels despite the sequence similarity of the two proteins. The amino acid sequences of the two proteins are 65% identical. *Efe*-AfpA and PAF were previously found to differ in activity against *Cl. jacksonii*, the causal agent of dollar spot disease on turfgrasses [25]. *Efe*-AfpA inhibited the growth of *Cl. jacksonii* but PAF had no inhibitory activity against the pathogen. Antifungal proteins have been identified from numerous fungal species and differences in the activity of other similar antifungal proteins have previously been reported [43,44]. Here, both *Efe*-AfpA and PAF had activity against all the Ascomycete fungi tested but the level of inhibition varied. *Efe*-AfpA had some activity against the Basidiomycete *L. fuciformis* whereas PAF had no activity. Neither protein had activity against the Basidiomycete *R. solani*.

The differences in activities of *Efe*-AfpA and PAF against the pathogens tested may reflect the different environments in which *E. festucae* and *Pe. chrysogenum* exist and the roles of these antifungal proteins in the biology of two fungi. *E. festucae* is a fungal endophyte of grasses and does not exist in nature independently of its grass host. PAF was originally reported from *Pe. chrysogenum* [9], which is commonly found in indoor environments and is a food spoilage fungus [45]. It is unknown if the antifungal activities of the two proteins are their main functions. If so, their differences in activity could be the result of evolutionary changes resulting from different competing fungi present in their environments. However, additional functions for both *Efe*-AfpA and PAF have been suggested, which could also contribute to the sequence differences that result in activity differences. *Efe*-AfpA was suggested to be critical for the interaction of the fungus with its host grass [46]. PAF was proposed to have roles in conidiation and autophagy [47,48]. AfpB from *Pe. digitatum* is another similar antifungal protein, but is also active against its parent strain. AfpB may play a role in regulating the *Pe. digitatum* population, as well as other fungal populations [49].

New strategies are needed to combat plant pathogens and the development of antifungal proteins could provide some alternatives or complements to traditional fungicides. The data presented here indicate that specific antifungal proteins could be developed to target particular plant pathogens. Since *Efe*-AfpA had activity against numerous other important plant pathogens, in addition to its activity against *Cl. jacksonii*, it could be developed to target a broader range of destructive pathogens in both turfgrass and agronomic systems.

## Figures and Tables

**Table 1:** Minimal and maximum inhibitory concentrations of *Efe*-AfpA and PAF against fungal plant pathogens.

	Minimal Inhibitory Concentration ^1^				Maximum Inhibitory Concentration ^2^			
	*Efe*-AfpA		PAF		*Efe*-AfpA		PAF	
Organism	µg mL^−1^	% Inhibition	µg mL^−1^	% Inhibition	µg mL^−1^	% Inhibition	µg mL^−1^	% Inhibition
Ascomycetes								
*B. cinerea*	0.6	94.4	−	−	0.6	94.4	0.6	89.6
*Co. cereale*	1.2	92.9	40	95.6	40	96.6	100	96.4
*Cr. parasitica*	0.5	98.8	0.5	100	0.5	98.8	0.5	100
*F. graminearum*	10	91	−	−	100	98.7	100	75.7
*P. oryzae*	−	−	−	−	100	87.1	20	75.3
Basidiomycetes								
*L. fuciformis*	−	−	−	−	100	46.5	−	−
*R. solani*	−	−	−	−	−	−	−	−

^1^ The minimal inhibitory concentration is defined as the concentration needed for 90% inhibition. “−” indicates activity did not result in 90% inhibition at any of the concentrations tested. ^2^ The maximum inhibitory concentration is defined as the highest inhibition obtained at any concentration tested. “−” indicates activity did not result in inhibition at any of the concentrations tested.

## Data Availability

All data supporting the findings of this study are available within the paper and within its Supplementary Materials published online.

## Supplementary Materials

The following supporting information can be downloaded at: https://www.mdpi.com/article/10.3390/microorganisms11040828/s1, Figure S1: Effect of *Efe*-AfpA and PAF on *Botrytis cinerea* growth; Figure S2: Effect of *Efe*-AfpA and PAF on *Colletotrichum cereale* growth; Figure S3: Effect of *Efe*-AfpA on *Cryphonectria parasitica* EP155 growth; Figure S4: Effect of PAF on *Cryphonectria parasitica* EP155 growth; Figure S5: Effect of *Efe*-AfpA and PAF on *Fusarium graminearum* Ph1 growth; Figure S6: Effect of *Efe*-AfpA and PAF on *Pyricularia oryzae* growth; Figure S7: Effect of *Efe*-AfpA on *Laetisaria fuciformis* growth; Figure S8: Effect of PAF on *Laetisaria fuciformis* growth; Figure S9: Effect of *Efe*-AfpA on *Rhizoctonia solani* growth; Figure S10: Effect of PAF on *Rhizoctonia solani* growth.
