# Supplementary material for: The Epichloë festucae Antifungal Protein Efe-AfpA Has Activity against Numerous Plant Pathogens
Source: Microorganisms. 2023 Mar 24;11(4):828. doi: 10.3390/microorganisms11040828 (PMC10145699; doi:10.3390/microorganisms11040828)
Supplement: Supplementary file 1 [file microorganisms-11-00828-s001.zip › microorganisms-2272247-supplementary.pdf]

The *Epichloë festucae* Antifungal Protein *Efe*-AfpA Has Activity Against Numerous Plant Pathogens

Patrick A. Fardella, Bruce B. Clarke, and Faith C. Belanger

Supplementary Materials

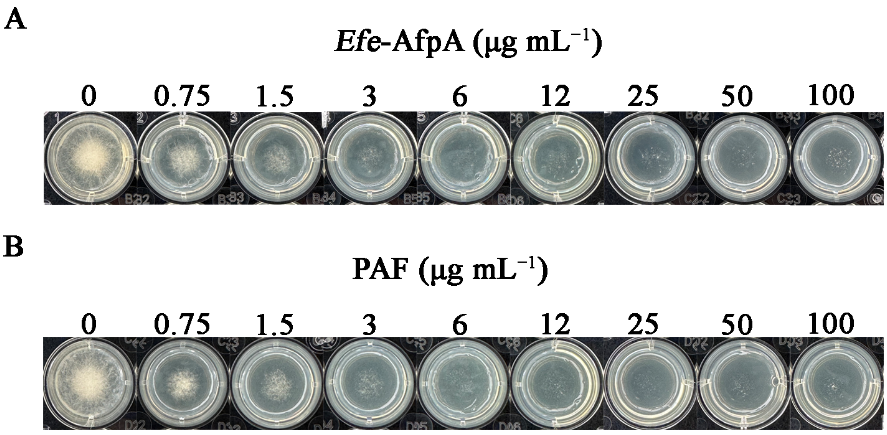

Figure S1. Effect of *Efe*-AfpA and PAF on *Botrytis cinerea* growth. *B. cinerea* spores were plated onto PDA amended with increasing concentrations of the antifungal proteins and photographed after 72 h at room temperature.

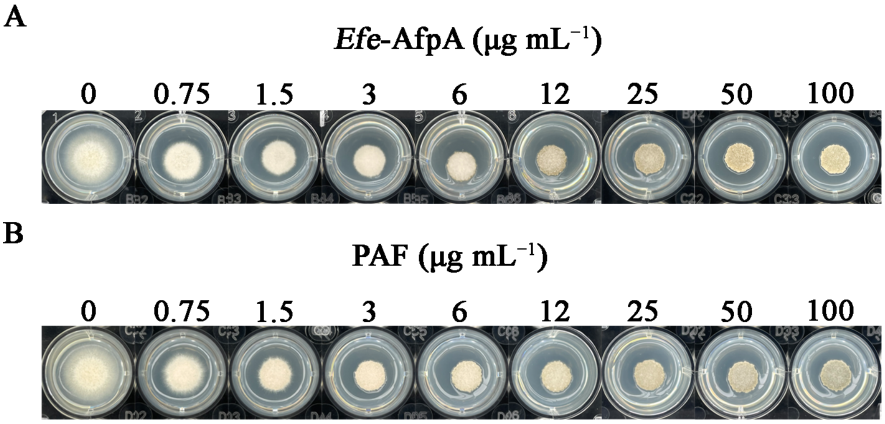

Figure S2. Effect of *Efe*-AfpA and PAF on *Colletotrichum cereale* growth. *Co. cereale* spores were plated onto PDA amended with increasing concentrations of the antifungal proteins and photographed after 72 h at room temperature.

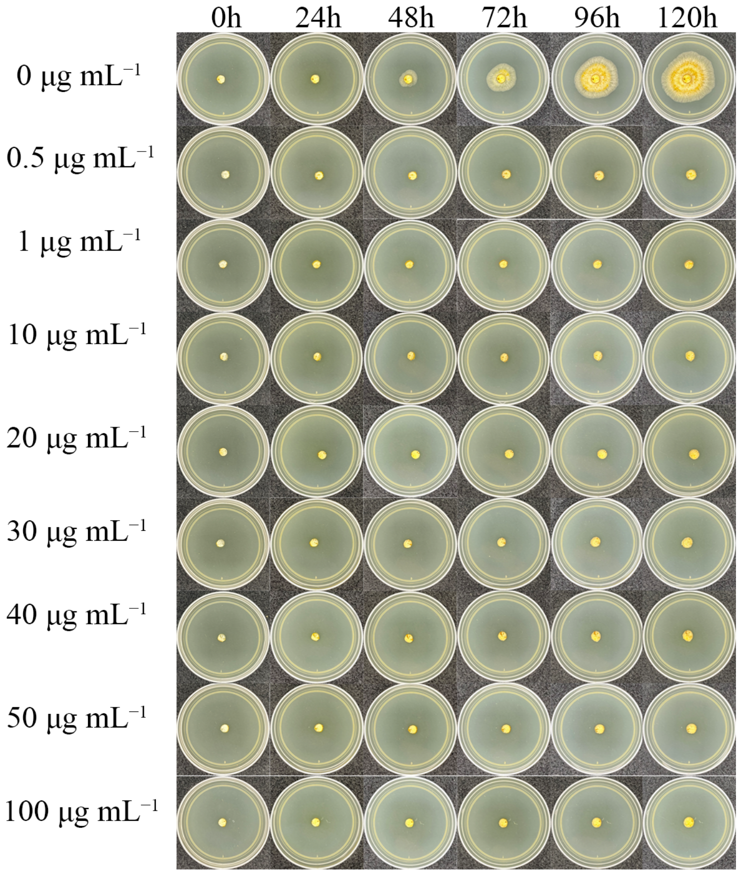

Figure S3. Effect of *Efe*-AfpA on *Cryphonectria parasitica* EP155 growth. *Cr. parasitica* mycelial plugs were subcultured onto PDA amended with increasing concentrations of *Efe*-AfpA and incubated at room temperature. The colony diameters were measured daily.

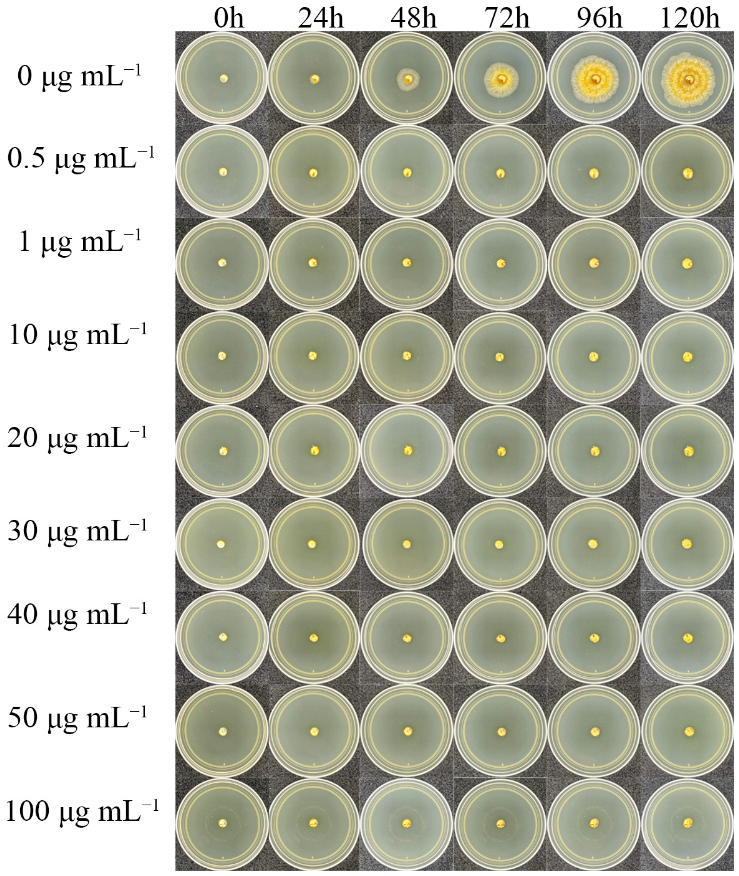

Figure S4. Effect of PAF on *Cryphonectria parasitica* EP155 growth. *Cr. parasitica* mycelial plugs were subcultured onto PDA amended with increasing concentrations of PAF and incubated at room temperature. The colony diameters were measured daily.

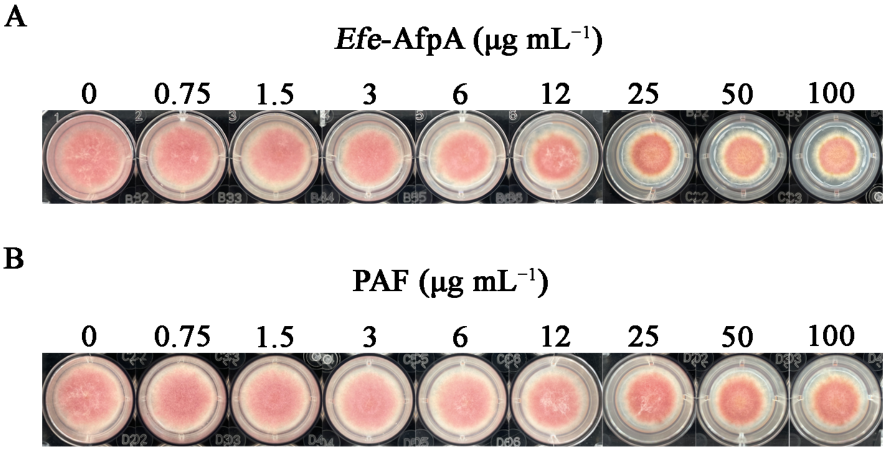

Figure S5. Effect of *Efe*-AfpA and PAF on *Fusarium graminearum* PH1 growth. *F. gramineaerum* spores were plated onto PDA amended with increasing concentrations of the antifungal proteins and photographed after 72 h at room temperature.

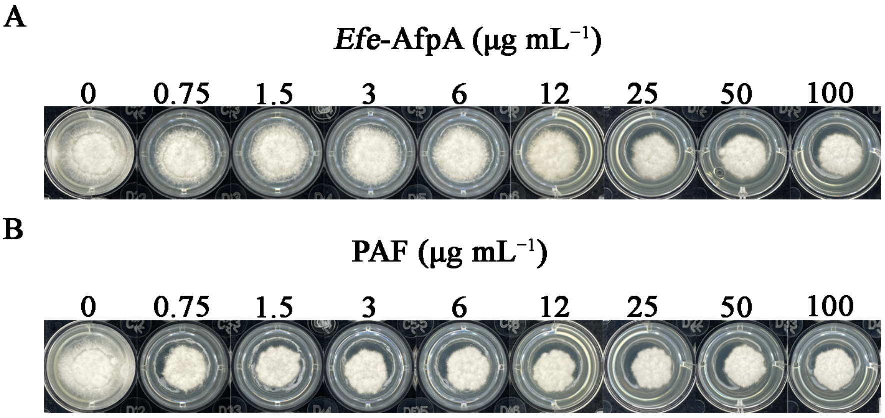

Figure S6. Effect of *Efe*-AfpA and PAF on *Pyricularia oryzae* growth. *Py. oryzae* spores were plated onto PDA amended with increasing concentrations of the antifungal proteins and photographed after 96 h at room temperature.

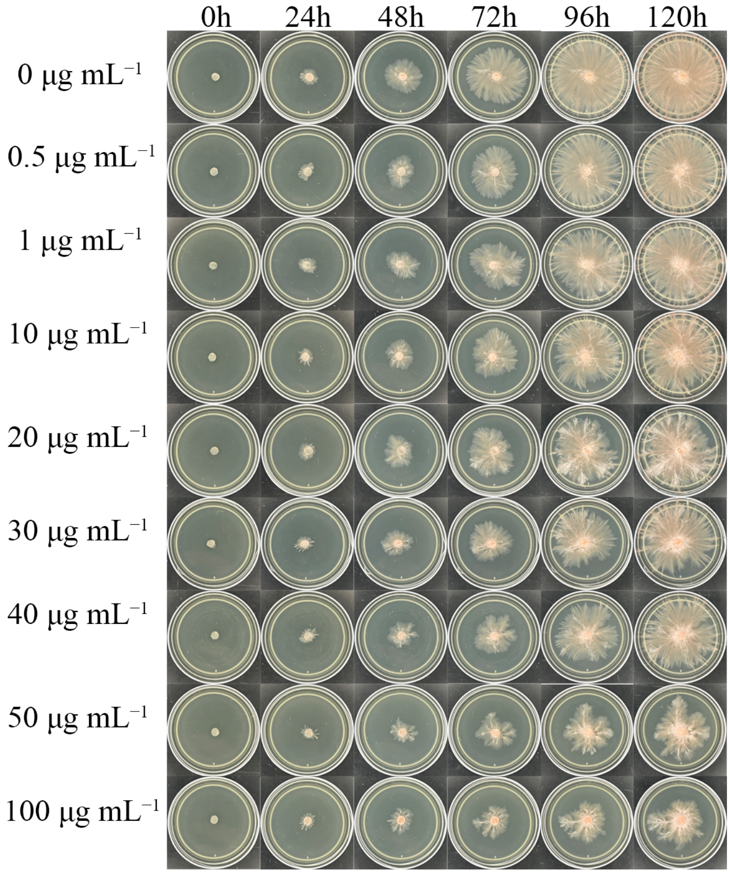

Figure S7. Effect of *Efe*-AfpA on *Laetisaria fuciformis* growth. *L. fuciformis* mycelial plugs were subcultured onto PDA amended with increasing concentrations of *Efe*-AfpA and incubated at room temperature. The colony diameters were measured daily.

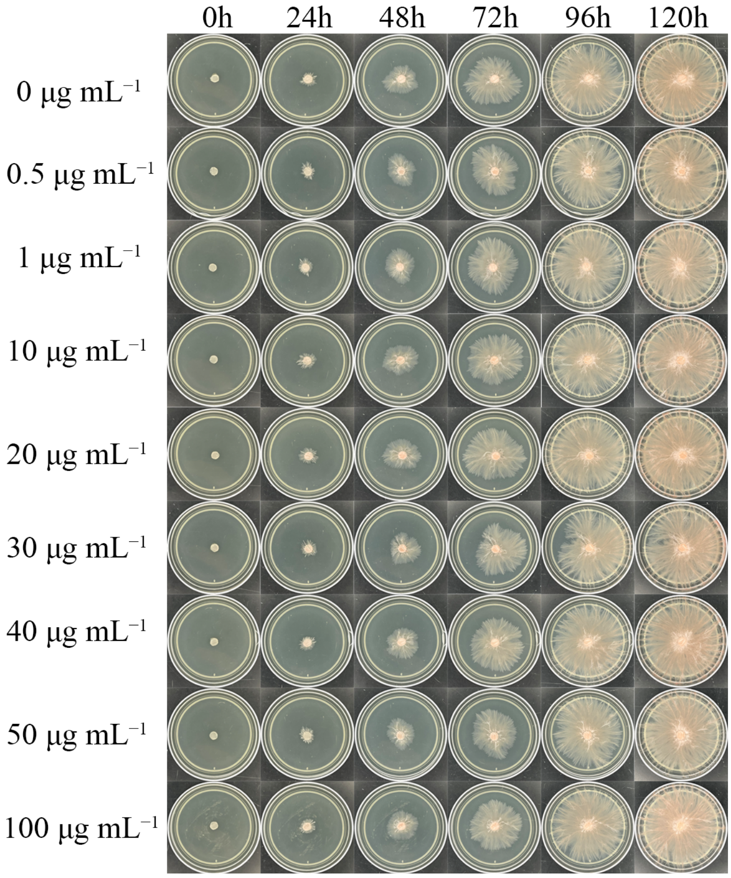

Figure S8. Effect of PAF on *Laetisaria fuciformis* growth. *L. fuciformis* mycelial plugs were subcultured onto PDA amended with increasing concentrations of PAF and incubated at room temperature. The colony diameters were measured daily.

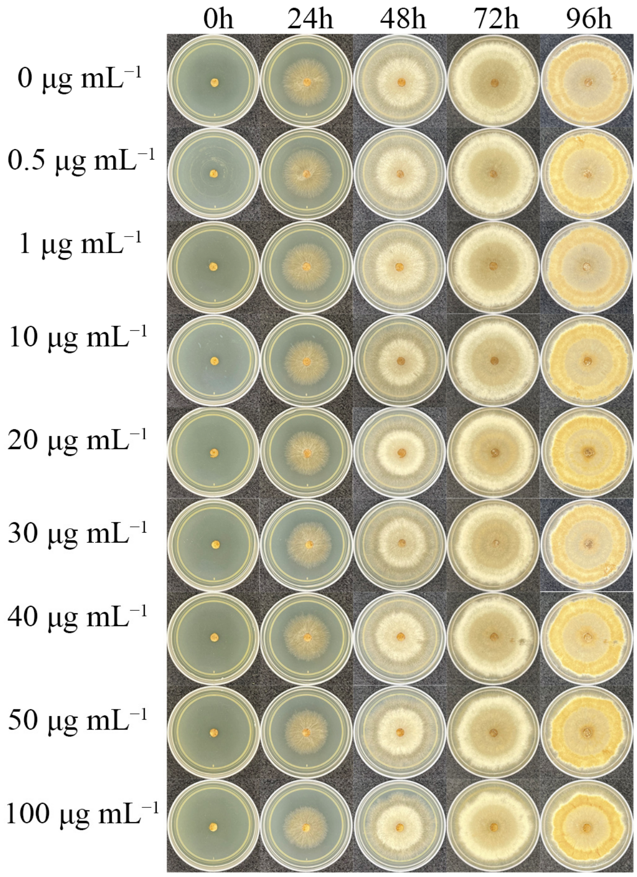

Figure S9. Effect of *Efe*-AfpA on *Rhizoctonia solani* growth. *R. solani* mycelial plugs were subcultured onto PDA amended with increasing concentrations of *Efe*-AfpA and incubated at room temperature. The colony diameters were measured daily.

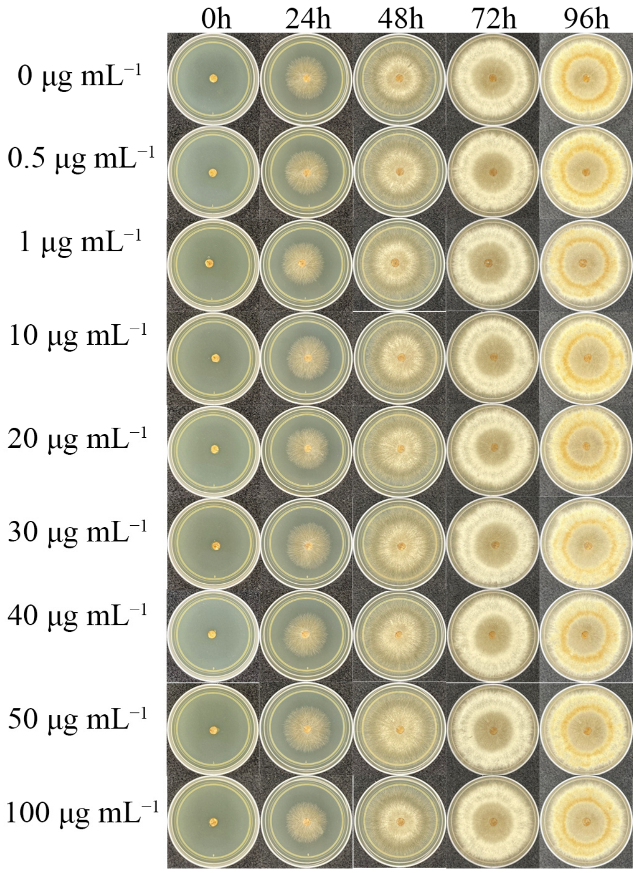

Figure S10. Effect of PAF on *Rhizoctonia solani* growth. *R. solani* mycelial plugs were subcultured onto PDA amended with increasing concentrations of PAF and incubated at room temperature. The colony diameters were measured daily.
